# Plasma exchange for COVID‐19 thrombo‐inflammatory disease

**DOI:** 10.1002/jha2.140

**Published:** 2020-11-30

**Authors:** Nishkantha Arulkumaran, Mari Thomas, David Brealey, Ferras Alwan, Deepak Singh, Michael Lunn, Anna Welch, Samuel Clark, Eamon Raith, Ugan Reddy, Ryan Low, David Leverett, Mervyn Singer, Marie Scully

**Affiliations:** ^1^ Intensive Care Unit University College London Hospitals NHS Foundation Trust London UK; ^2^ Bloomsbury Institute of Intensive Care Medicine University College London London UK; ^3^ Department of Haematology University College London Hospitals NHS Foundation Trust and Cardiometabolic Programme‐NIHR UCLH/UC BRC London UK; ^4^ Department of Haematology University College London Hospitals NHS Foundation Trust London UK; ^5^ Special Coagulation UCLH‐HSL London UK; ^6^ National Hospital for Neurology and Neurosurgery University College London Hospitals NHS Foundation Trust London UK

**Keywords:** COVID‐19, inflammation, lymphopenia, plasma exchange, thrombosis

## Abstract

Severe COVID‐19 disease is a hyperinflammatory, pro‐thrombotic state. We undertook plasma exchange (PEX) to determine its effects on organ function and thrombo‐inflammatory markers.

Seven critically ill adults with severe COVID‐19 respiratory failure (PaO_2_:FiO_2_ ratio < 200 mm Hg) requiring invasive or noninvasive ventilatory support and elevated thrombo‐inflammatory markers (LDH >800 IU/L and D‐dimer >1000 μg/L (or doubling from baseline) received PEX, daily, for a minimum of 5 days. No other immunomodulatory medications were initiated during this period. Seven patients matched for age and baseline biochemistry were a comparator group.

Coagulation screening revealed no evidence of coagulopathy. However, von Willebrand Factor (VWF) activity, antigen and VWF antigen: ADAMTS13 ratio, Factor VIII and D‐dimers were all elevated. Following 5 days of PEX, plasma levels of all the above, and ferritin levels, were significantly reduced (*P* < .05) while lymphocyte counts normalized (*P* < .05). The P_a_O_2_:FiO_2_ ratio increased from a median interquartile range (IQR) of 11.6 (10.8‐19.7) kPa to 18.1 (16.0‐25.9) kPa (*P* < .05). Similar improvements were not observed in controls. Acute kidney injury (AKI) occurred among five patients in the control arm but not in patients receiving PEX.

PEX improved oxygenation, decreased the incidence of AKI, normalized lymphocyte counts and reduced circulating thrombo‐inflammatory markers including D‐Dimer and VWF Ag:ADAMTS13 ratio.

## 1 INTRODUCTION

Severe COVID‐19 disease is associated with a hyperinflammatory, pro‐thrombotic state, marked endothelial activation, and a high mortality [1]. The incidence of clinical thromboembolic complications in critically ill patients with COVID‐19 is much higher than with traditional causes of acute respiratory distress syndrome (ARDS) [2]. Furthermore, histopathological studies identify thrombi as a major feature in post‐mortem COVID‐19 lung specimens [3, 4].

Inflammation results in endothelial activation and release of von Willebrand Factor (VWF) [5], a key component of primary hemostasis. Breakdown of VWF into smaller multimers is predominantly mediated by the metalloproteinase, ADAMTS13 (a disintegrin‐like and metalloprotease with thrombospondin type 1 *motif 13*). We noted a high VWf Ag:ADAMTS13 ratio in our COVID‐19 patients, which may contribute to the inflammatory, prothrombotic phenotype, comparable to parameters seen in thrombotic microangiopathy (TMA), with evidence of micro and macro thrombosis, primarily affecting the lungs. We therefore performed a preliminary exploration of the impact of plasma exchange (PEX), a therapy successfully used in TMA conditions [6], on inflammatory and coagulation markers and organ function in a critically ill cohort of COVID‐19 patients with ARDS.

## 2 METHODS

Critically ill adults (≥18 years) patients with proven COVID‐19 disease had markers of coagulation measured on admission to the intensive care unit (ICU). Subsequently, a prospective case‐controlled, non‐blinded descriptive study was performed to assess the effectiveness and safety of PEX. Institutional agreement was confirmed, and written informed consent was obtained from patients or their next‐of‐kin.

Respiratory failure criteria for treatment included bilateral infiltrates on chest imaging, worsening respiratory function, based on a ratio of arterial oxygen partial pressure to fractional inspired oxygen (P_a_O_2_:FiO_2_ ratio) <200 mm Hg (26.7 kPa) despite treatment with noninvasive continuous positive airway pressure (CPAP) ventilation or invasive mechanical ventilation invasive mechanical ventilation for at least 24 hours. Laboratory criteria included elevated plasma lactate dehydrogenase (LDH) > 800 IU/L or a doubling from baseline, and increased D‐dimer >1000 μg/L or a doubling from baseline. Patients <18 years, pregnant, actively bleeding or unsuitable for invasive mechanical ventilation were excluded.

Control patients were admitted at the same time as patients undergoing PEX and met the inclusion criteria for PEX with similar clinical and biochemical parameters.

Demographic, clinical and laboratory data were collected for all patients, including platelet, lymphocyte counts, C‐reactive protein (CRP), and creatinine. Patients undergoing PEX had additional blood tests including ferritin, LDH, and D‐dimer, ADAMTS 13 activity (using a fluorescence resonance energy transfer‐VWF73 assay modified from Kokame et al) [7], factor VIII, von Willebrand activity and antigen levels. Protein C, protein S, and antithrombin III levels and the VWD screen were measured on CS2500 analysers (Sysmex, Milton Keynes, UK). Serum cytokines (interleukin‐1 beta [IL‐1ß], IL‐6, IL‐10, tumor necrosis factor‐alpha [α]) were measured using multiplex assays (V‐plex, Mesoscale Discovery, Rockville, MD). Baseline blood tests were taken within 24 hours of initiation of the first PEX and repeated after five daily sessions.

Patients receiving PEX treatment had three‐liter single volume daily, with Octaplas LG (Octapharma, Manchester, UK) using a Spectra Optia Apheresis system (Terumo BCT, Lakewood, CO). Citrated and serum samples were taken before and after PEX. Clinical and laboratory assessment of response and complications were undertaken daily. Routine clinical data were collected from hospital electronic healthcare records. All patients who underwent PEX received intermediate dose low molecular weight heparin as routine thromboprophylaxis.

The equivalent of day 0 PEX treatment for the control arm was taken as day 4 or 5 from hospital admission, as the median time from hospital admission to PEX was 4.5 days. Control cases had standard thromboprophylaxis as per hospital guidelines, unless a thromboembolic event had been confirmed, in which case they received treatment doses LMWH.

All statistical analyses were carried out using GraphPad Prism (v8, GraphPad Software, San Diego, CA). Descriptive data, expressed as median (interquartile range), were generated on demographics, clinical features, interventions, and outcomes. Differences in medians before and after PEX were assessed using Wilcoxon matched‐pairs signed rank test. Statistical significance is regarded when *P*‐values were below .05 (two‐sided).

## 3 RESULTS

Levels of VWF activity, VWF antigen, VWF antigen:ADAMTS13 ratio, and D‐dimers were significantly increased in critically ill patients with COVID‐19 disease (Table 1). However, other markers of coagulation, including endogenous anticoagulant levels, were all within the normal range. Baseline demographics and laboratory parameters were similar between PEX and control group patients (Table 2).

**TABLE 1:** Coagulation factors in patients with COVID‐19 disease and ARDS

	Reference range	Value median (IQR)
**PT (s)**	10‐12	11.4 (10.8‐11.4)
**APTT (s)**	25‐37	34 (31‐42)
**Platelets (10^9^/L)**	150‐400	238 (205‐278)
**Fibrinogen (g/L)**	1.5‐4.0	4.96 (3.94‐8.02)
**Antithrombin activity (IU/mL)**	0.66‐1.24	0.88 (0.80‐1.10)
**Protein C (IU/mL)**	0.65‐1.35	0.84 (0.68‐1.41)
**Protein S (IU/mL)**	0.70‐1.40	0.86 (0.55‐1.00)
**ADAMTS13 (%)**	60‐146	73 (65‐89)
**VWF activity (IU/mL)**	0.5‐1.87	2.9 (1.8‐4.8)*
**VWF antigen (IU/mL)**	0.5‐1.6	3.3 (1.9‐4.9)*
**VWF Ag/ADAMTS13 ratio**	0.34‐2.7	4.0 (2.8‐5.7)*
**Factor VIII (IU/mL)**	0.5‐2.0	3.9 (2.0‐4.9)*
**D‐ dimer (ug/L FEU)**	0‐550	1700 (690‐10330)*
**LDH (U/L)**	90‐235	466 (399‐607)*

A summary of coagulation parameters from patients with severe COVID19 in intensive care, including those intubated and ventilated and noninvasive ventilation.

Abbreviations: ADAMTS13, a disintegrin‐like and metalloprotease with thrombospondin type 1 *motif 13*; APTT, activated partial thromboplastin time; LDH, lactate dehydrogenase; PT, prothrombin time; VWF, von willerbrand factor.

* Elevated above normal range.

**TABLE 2:** Baseline characteristics of patients receiving plasma exchange and control patients

	Control patients	PEX patients
**Age (years)**	57 (49‐66)	53 (45‐60)
**Gender**	M:F = 6:7	M:F = 3:4
**Ethnicity**	Caucasian: 5 South‐ Asian: 2	Caucasian: 3 East‐ Asian: 1 Middle‐Eastern: 2 African: 1
**Weight (kg)**	80 (77‐92)	79 (76‐99)
**Symptoms to hospital admission (days)**	7 (4‐11)	9 (6‐11)
**Baseline biochemistry**		
**CRP (mg/L)**	230 (203‐335)	300 (128‐349)
**Lymphocyte (/10^9^)**	0.9 (0.6‐1.0)	0.91 (0.53‐1.10)
**Creatinine (μmol/L)**	64 (62‐84)	54 (42‐68)
**PaO_2_:FiO_2_ ratio (mm Hg)**	119 (82‐127)	87 (81‐148)
**Mechanical ventilation during time of PEX**	6 of 7 (86%)	6 of 7 (86%)

A comparison of the demographic features between those patients who received 5 days of PEX and comparable control patients on the intensive care unit at this time.

Continuous data represent median and interquartile range.

Seven patients were formally approached for PEX of whom one declined. One patient completed only two cycles of PEX as he was transferred for extracorporeal membrane oxygenation following development of a large pneumomediastinum unrelated to PEX treatment. This patient was excluded from subsequent analyses. All other patients completed five cycles. A further two patients underwent PEX on compassionate grounds. All patients were followed‐up for a minimum of 28 days. Demographics are shown in Table S1. All patients had good baseline functional status before COVID‐19 infection. The median duration from symptom onset was 9 (6‐11) days, and time from hospital admission to PEX was 3 (2‐5) days in the initial five patients, and 34 days in the two patients treated on compassionate grounds. Neither patients in the PEX or control groups received steroids or other immunomodulatory agents.

Of the initial five patients who received PEX, three were receiving noninvasive CPAP and two invasive mechanical ventilation. Within 24 hours of commencement of PEX, a further two patients required invasive mechanical ventilation. The two patients treated on compassionate grounds were receiving invasive mechanical ventilation. No major adverse events, including thrombotic or bleeding episodes, occurred during PEX.

**FIGURE 1:**
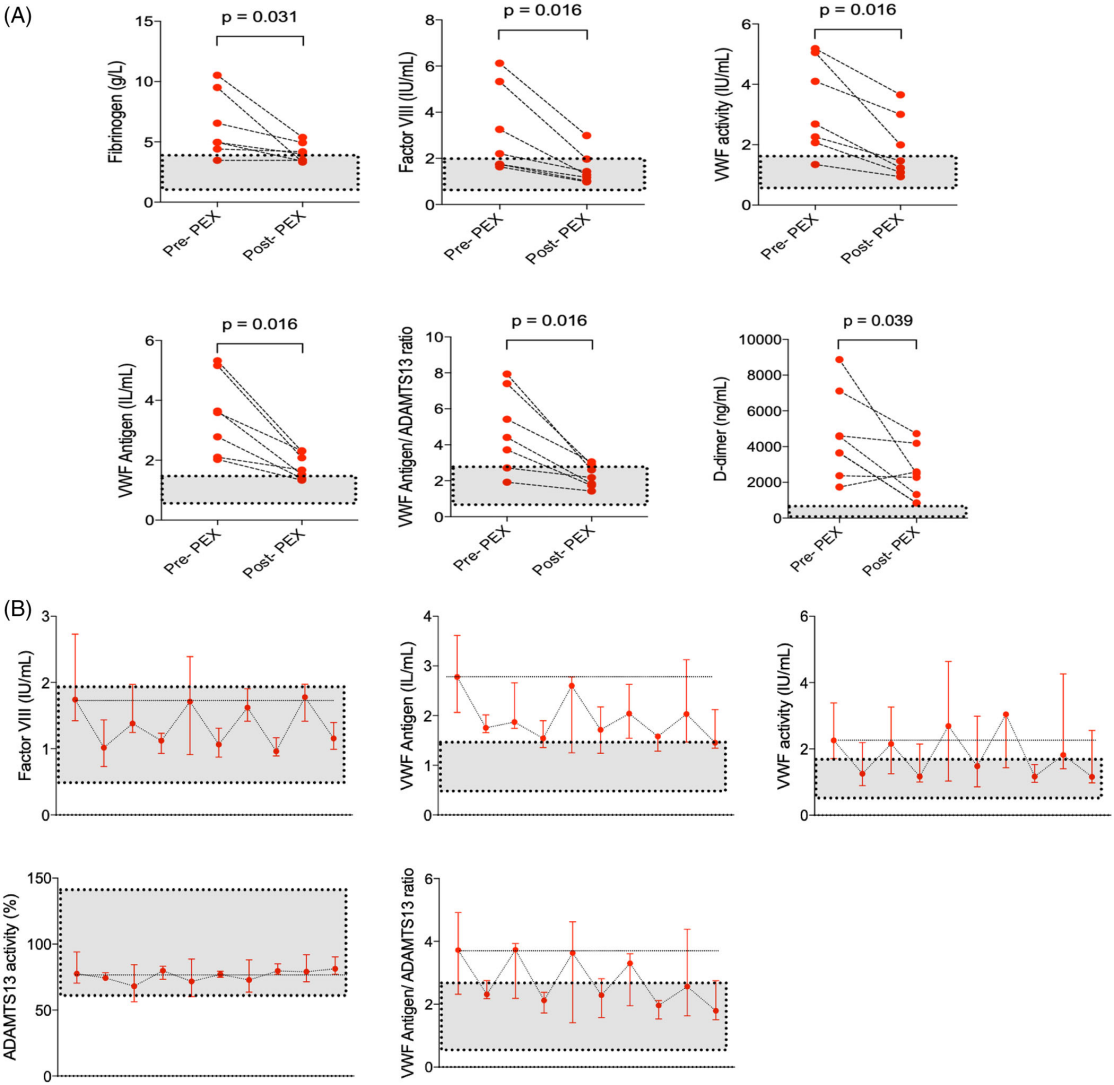
**A and B,** Comparison of coagulation data of patients before and following five courses of plasma exchange. Grey areas highlight normal ranges (n = 7) Abbreviation: PEX, plasma exchange.

**FIGURE 2:**
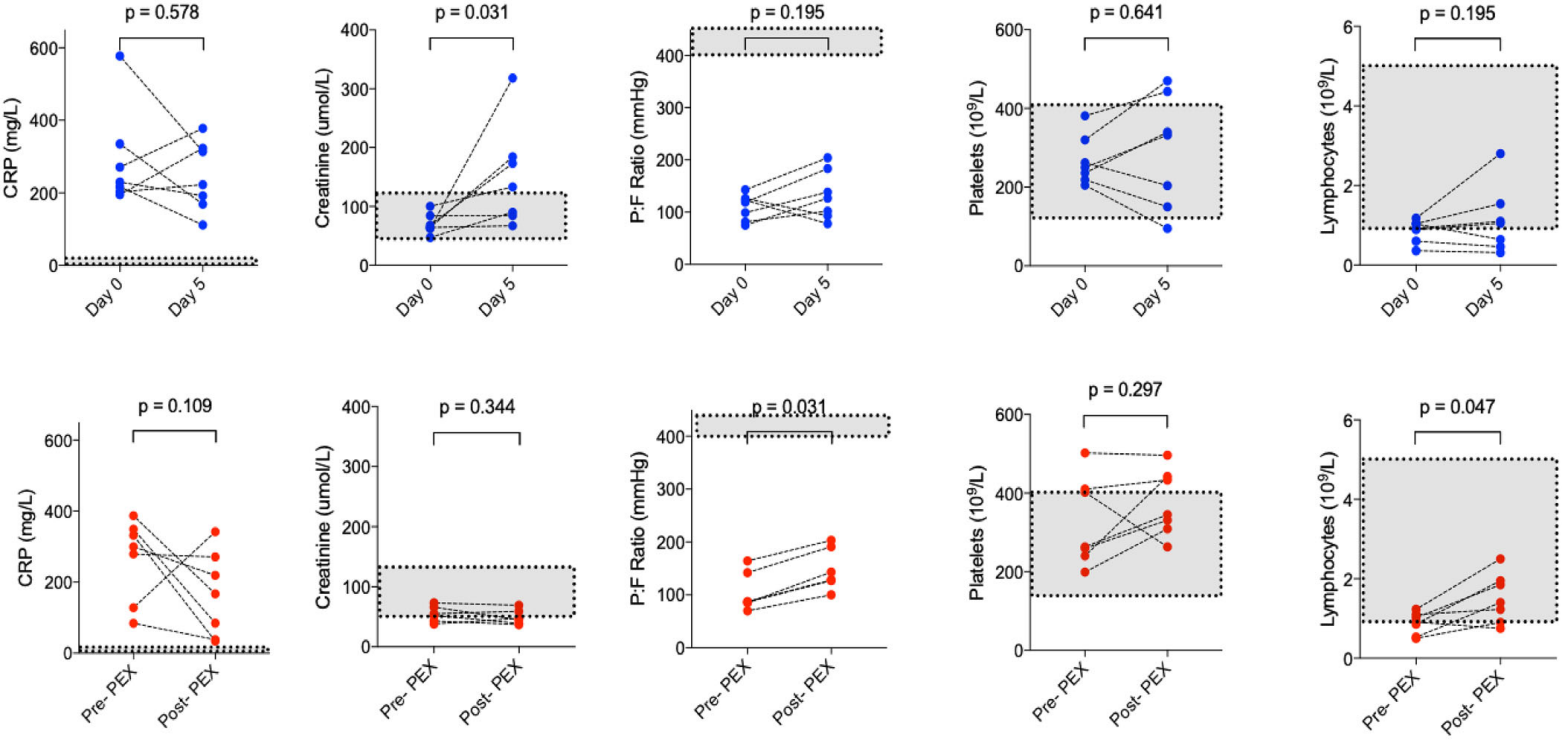
Comparison of clinical and biochemical data of control patients and patients before and following five courses of plasma exchange. Top panel in blue: control patients. Bottom panel in red: PEX patients. Gray areas highlight normal ranges. (n = 7) apart from P_a_O_2_:FiO_2_ ratio in PEX group (n = 6) and serum creatinine (n = 6) Abbreviation: PEX, plasma exchange.

**FIGURE 3:**
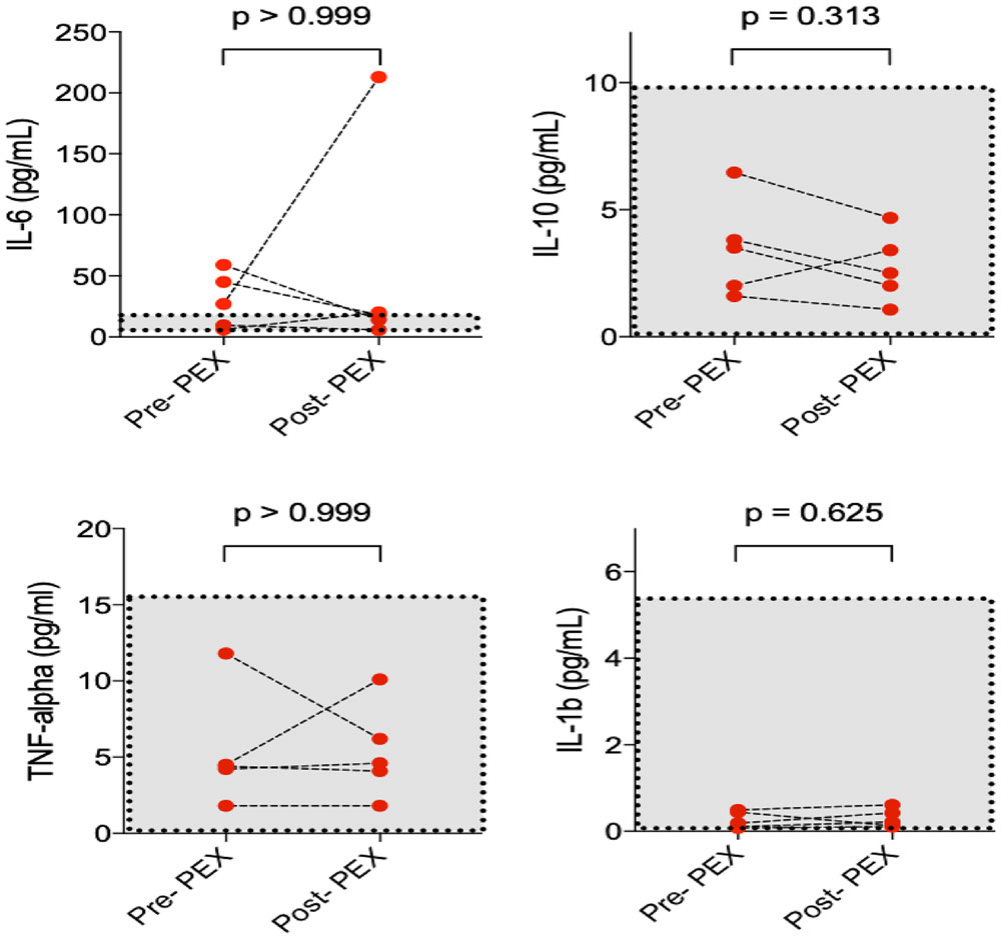
Comparison of serum cytokines before and following five courses of plasma exchange (n = 5). Cytokines measured using a multiplex assay, from PRE the 1st PEX and samples taken post the final PEX on day 5. The increased IL‐6 post‐PEX in one case represents an increased CRP and severe sepsis. There was a trend for decrease in cytokines, but this was not significant. Other than IL‐6, the remaining cytokines were within the normal range

At the end of the 5‐day course, PEX reduced VWF antigen and activity, VWF antigen/ADAMTS13 ratio and Factor VIII levels; all *P* < .05 (Figure 1A and B; Table 3). ADAMTS13 activity remained unchanged over this period (*P* = .375). There was a significant fall of inflammatory markers including ferritin (*P* = .016) and D‐dimers (*P* = .039), and improvement in lymphopenia (*P* = .047) (Figure 2; Table 3). There was no statistically significant change in CRP (*P* = .109), albeit this was skewed by one patient who developed a secondary *Klebsiella pneumoniae* sepsis. No significant changes were seen in serum cytokines or platelet count (*P* = .297). Serum cytokines were not elevated at baseline, and PEX had no effect on circulating levels of serum cytokines (Figure 3).

**TABLE 3:** Comparison of clinical, biochemical, and coagulation data of patients before and following five courses of plasma exchange

	Reference range	Pre‐plasma exchange median (IQR)	Post‐plasma exchange median (IQR)	*P*‐value
**P:F ratio (mm Hg)**	>53	11.5 (10.8‐19.7)	18.1 (16.0‐24.9)	**.031**
**Creatinine (μmol/L)**	49‐90	54 (42‐68)	46 (39‐62)	.344
**Platelets (10^9^/L)**	150‐400	263 (240‐410)	345 (309‐442)	.297
**Lymphocyte (10^9^/L)**	1.0‐4.8	0.91 (0.53‐1.10)	1.40 (0.90‐1.95)	**.047**
**D‐ dimer (ug/L FEU)**	0‐550	4110 (2690‐6483)	2385 (968‐3790)	**.039**
**LDH (IU/L)**	90‐235	431 (399‐595)	426 (308‐446)	.188
**CRP (mg/L)**	0‐5	300 (128‐349)	167 (38‐271)	.109
**Ferritin (ng/mL)**	10‐300	1003 (514‐3373)	568 (331‐685)	**.016**
**ADAMTS13 activity (%)**	60‐146	75 (66‐83)	79 (77‐83)	.375
**VWF activity (IU/mL)**	0.5‐1.87	2.68 (2.07‐5.05)	1.46 (1.08‐3.00)	**.016**
**VWF antigen (IU/mL)**	0.5‐1.6	3.6 (2.1‐5.2)	1.7 (1.4‐2.3)	**.016**
**VWF Antigen/ADAMTS13 ratio**	0.34‐2.7	4.4 (2.7‐7.4)	2.2 (1.7‐2.9)	**.016**
**Factor VIII (IU/mL)**	0.5‐2.0	2.2 (1.7‐5.3)	1.3 (1.0‐2.0)	**.016**
**Fibrinogen (g/L)**	1.5‐4.0	4.96 (4.41‐9.50)	3.98 (3.39‐4.93)	**.031**
**TNF‐alpha (pg/mL)**	0‐16	4.4 (3.0‐8.2)	4.6 (3.0‐8.2)	>.999
**IL‐1b (pg/mL)**	0‐5	0.19 (0.10‐0.47)	0.22 (0.12‐0.52)	.625
**IL‐6 (pg/mL)**	5‐15	27 (8‐52)	18 (10‐117)	>.999
**IL‐10 (pg/mL)**	0‐10	3.5 (1.98‐5.1)	2.5 (1.5‐4.0)	.313

A summary of the impact of PEX on thrombotic, inflammatory, and cytokine parameters and organ function measured before initiation of and on completion of PEX.

n = 7; apart from P:F ratio (n = 6); LDH (n = 5); serum cytokines (n = 5), and serum creatinine (n = 6).

Data are expressed as median and interquartile range.

The PaO_2_:FiO_2_ ratio increased in all patients from 87 (81‐148) mm Hg at baseline to 136 (120‐194) mm Hg after 5 days of PEX (Figure 2; Table 3). No patient developed acute kidney injury (AKI) during PEX treatment; the one patient requiring renal replacement therapy at baseline had this discontinued at day 4.

In contrast, there was no significant fall in CRP, recovery of lymphocytes, or improvement in PaO_2_:FiO_2_ ratio among control group patient (Figure 2). Furthermore, there was an increase in the serum creatinine (*P* < .05), with five patients in the control group developing new onset AKI.

## 4 DISCUSSION

Severe infection with COVID‐19 presents a unique thrombo‐inflammatory syndrome that remains incompletely characterized. The very high reported incidence of arterial, venous and pulmonary thromboembolism detected by imaging [2], and the widespread pulmonary macro‐ and microthrombi noted at post‐mortem implicates the prothrombotic component as a major factor underlying the pathophysiology of this disease [3, 4].

COVID‐19 shares features of TMA but displays a distinct phenotype. Indeed, we found a raised VWF, elevated Factor VIII and an increased VWF antigen:ADAMTS13 ratio indicative of a prothrombotic predisposition. Conversely, ADAMTS13 activity was normal, and there was no thrombocytopenia. Levels of other coagulation factors and endogenous anticoagulants such as antithrombin, protein C and protein S were remarkably normal, especially given the likelihood of markedly raised fibrin generation as suggested by high levels of D‐dimers. Fibrinogens were high, behaving as an acute phase marker. These coagulation abnormalities appear unique to COVID‐19 disease. There were no consumptive features as seen in disseminated intravascular coagulation (DIC), which might be expected with the continuous, severe inflammatory‐thrombotic process.

PEX restores hemostatic balance and reduces levels of circulating inflammatory markers in a range of TMA. There was therefore a compelling rationale to explore its utility in COVID‐19. We therefore undertook a limited case‐controlled study to determine safety and feasibility, and its impact on thrombo‐inflammatory markers and respiratory parameters. We were able to demonstrate normalization of prothrombotic and acute phase response markers, such as ferritin, fibrinogen, and CRP following 5 days of PEX. The VWF Ag:ADAMTS13 ratio has been highlighted in other thrombotic conditions with higher ratios being associated with inferior outcomes in stroke and cardiac disease [8, 9]. Lymphopenia and elevated D‐dimers levels, poor prognostic risk factors in COVID‐19, were also reversed [10]. Notably, circulating levels of proinflammatory cytokines were unaffected, but these were not particularly high at baseline, particularly in relation to other causes of ARDS such as bacterial sepsis [11]. Our finding of low levels of circulating cytokines is in keeping with other studies in COVID‐19 patients, suggesting the description of "cytokine storm" is a misnomer in this condition.

Our finding of an improvement in PaO_2_:FiO_2_ ratio among all treated patients is of particular importance, as mortality in COVID‐19 is primarily associated with severe hypoxemic respiratory failure and histological features of pulmonary thrombosis. The fall in D‐dimers, as a marker of fibrinolytic activity, likely represents a reduction in fibrin clot formation. No safety concerns were highlighted during the course of PEX, in particular no bleeding or AKI.

Three patients deteriorated once PEX was stopped with worsening gas exchange and a recrudescence in inflammatory marker levels. This suggests that PEX may have dampened the flames transiently, but an underlying pro‐inflammatory focus remains, most likely within the lung. The two cases treated on compassionate grounds, which had both prolonged ICU stay and extended PEX therapy, further expand our understanding of severe later stage COVID‐19. It was anticipated that their ongoing severe ARDS picture represented a fibrotic stage of disease however, with PEX, their inflammatory markers improved as did their respiratory parameters and, in one case, renal function.

Despite promising results, we acknowledge a number of limitations. This was a small case controlled study. There was also variation in the timing of initiation of PEX within the disease course. The subset of patients who are most likely to benefit from PEX and the optimal duration of treatment remains unknown. A longer course, or a repeat course, could be envisaged, recognizing this may create logistic difficulties in terms of the number of patients who can be treated concurrently.

## 5 CONCLUSION

PEX in patients with severe COVID‐19 disease with ARDS was associated with improvements in oxygenation and reversal of the thrombo‐inflammatory markers and normalization of the lymphocytes, even when used late in the disease process. There is a positive impact on organ function and significant improvement in parameters defined as poor prognosis in severe COVID‐19 infection. This provides support for conduct of a formal randomized controlled trial.

## CONFLICT OF INTEREST

Mervyn Singer received speakers fees and honoraria for advisory boards from Alexion, Sanofi, Novartis, and Takeda. Mari Thomas received speakers fees and advisory board honoraria from Bayer and Sanofi. All authors have no conflict of interest to declare.

## AUTHOR CONTRIBUTIONS

Study concept and design: Arulkumaran, Thomas, Brealey, Mervyn Singer, and Marie Scully. Gathered data: Arulkumaran, Thomas, Alwan, Singh, Lunn, Welch, Low, Mervyn Singer, and Marie Scully. Performed study: Arulkumaran, Thomas, Brealey, Clark, Raith, Reddy, Leverett, Mervyn Singer, and Marie Scully. Drafting of the manuscript: Arulkumaran, Thomas, Brealey, Alwan, Singh, Lunn, Welch, Clark, Raith, Reddy, Low, Leverett, Mervyn Singer, and Marie Scully. Critical revision of the manuscript for important intellectual content: Arulkumaran, Thomas, Brealey, Alwan, Singh, Lunn, Welch, Clark, Raith, Reddy, Low, Leverett, Mervyn Singer, and Marie Scully.

## Supporting information


**Table S1** Detailed characteristics of patients receiving plasma exchange
