# Supplementary material for: Plasma exchange for COVID‐19 thrombo‐inflammatory disease
Source: EJHaem. 2020 Nov 30;2(1):26–32. doi: 10.1002/jha2.140 (PMC7754560; doi:10.1002/jha2.140)
Supplement: Supplementary file 1 — Table S1 Detailed characteristics of patients receiving plasma exchange [file JHA2-2-26-s001.docx]

**Supplementary Table 1**

Detailed characteristics of patients receiving plasma exchange

| Patient No | 1 | 2 | 3 | 4 | 5 | 6 | 7 |
| --- | --- | --- | --- | --- | --- | --- | --- |
| Age (Years) | 39 | 53 | 57 | 60 | 45 | 50 | 64 |
| Gender | F | F | M | F | M | M | M |
| Ethnicity | Middle Eastern | African | Middle Eastern | Caucasian | SE Asia | Caucasian | Caucasian |
| Weight (kg) | 97 | 105 | 70 | 78 | 78 | 90 | 80 |
| Co-morbid illness | Obesity  Asthma | Obesity | Nil | Asthma | Asthma | None | Previous DVT |
| Symptoms to hospital admission (days) | 12 | 3 | 9 | 6 | 11 | 11 | 7 |
| Hospital admission to PEX (days) | 6 | 2 | 5 | 5 | 4 | 34 | 34 |
| Days of PEX | 5 | 5 | 5 | 5 | 5 | 10 | 8 |
| Ventilation at PEX initiation | CPAP | MV | CPAP | CPAP | CPAP | IMV | IMV |

(M: Male; F: Female; DVT: deep vein thrombosis; PEX: plasma exchange; CPAP: continuous positive airway pressure; IMV: invasive mechanical ventilation)
